# Effects of renal denervation therapy on cardiac function and malignant arrhythmia in patients with reduced left ventricular ejection fraction and narrow QRS complexes treated with implantable cardioverter defibrillator

**DOI:** 10.3389/fphys.2022.938486

**Published:** 2022-08-12

**Authors:** Wei Yang, You-Long Xu, Jun-Qing Gao, Deng Shen, Min Li, Jing-Jing Fa, Ying Zhang, Rui Wang, Shu-Xin Hou, Wen-Ying Hu, Hui-Gen Jin, Zong-Jun Liu

**Affiliations:** ^1^ Department of Cardiology, Putuo Hospital, Shanghai Putuo District Central Hospital, Shanghai University of Traditional Chinese Medicine, Shanghai, China; ^2^ Department of Cardiology, Shanghai Putuo Central School of Clinical Medicine, Anhui Medical University/The Fifth School of Clinical Medicine, Anhui Medical University, Hefei, China

**Keywords:** heart failure, narrow QRS complex, renal denervation (RDN), malignant arrhythmia, randomized controlled trial

## Abstract

**Objective** **:** The purpose of this study was to explore the effects of renal denervation (RDN) on cardiac function and malignant arrhythmia in patients with reduced left ventricular ejection fraction (HFrEF) and narrow QRS treated with an implantable cardioverter defibrillator (ICD).

**Methods:** A total of 20 eligible HFrEF patients [left ventricular ejection fraction (LVEF) <40%] and narrow QRS complexes (QRS duration <120 ms) were randomized into either the ICD plus RDN group or the ICD only group during 17 April 2014 to 22 November 2016. Clinical data, including clinical characteristics, blood biochemistry, B-type natriuretic peptide, echocardiographic indexes, 6-min walk distance (6MWD), New York Heart Association (NYHA) classification, and count of ICD discharge events before and after the operation were analyzed. Patients were followed up for up to 3 years post ICD or ICD plus RDN.

**Results:** Baseline clinical data were comparable between the two groups. Higher LVEF (%) (mixed model repeated measure, *p* = 0.0306) (39.50% ± 9.63% vs. 31.20% ± 4.52% at 1 year; 41.57% ± 9.62% vs. 31.40% ± 8.14% at 3 years), systolic blood pressure (*p* = 0.0356), and longer 6MWD (*p* < 0.0001) as well as reduction of NYHA classification (*p* < 0.0001) were evidenced in the ICD plus RDN group compared to ICD only group during follow-up. Patients in the ICD plus RDN group experienced fewer ICD discharge events (2 vs. 40) and decreased diuretic use; rehospitalization rate (30% vs. 100%, *p* = 0.0031) and cardiogenic mortality rate (0% vs. 50%, *p* = 0.0325) were also significantly lower in the ICD plus RDN group than in the ICD only group during follow-up.

**Conclusion:** ICD implantation plus RDN could significantly improve cardiac function and cardiac outcome as well as increase exercise capacity compared to ICD only for HFrEF patients with narrow QRS complexes.

## Introduction

Heart failure (HF) patients with reduced LVEF (HFrEF) are associated with persistent symptoms and significant functional limitations (Sénior et al., 2016), high mortality and rehospitalization rates, poor quality of life, and high health care costs (Crespo-Leiro et al., 2016; Ponikowski et al., 2016; Sciatti et al., 2019). According to the 2012 European Society of Cardiology (ESC) Guidelines, cardiac resynchronization therapy (CRT) should be considered in patients in New York Heart Association (NYHA) functional class III or ambulatory class IV with QRS duration ≥120 ms and ejection fraction (EF) ≤35% (McMurray et al., 2012). The 2017 Canadian Cardiovascular Society Guidelines recommended CRT for patients in sinus rhythm with NYHA class II or III or ambulatory class IV HF despite optimal medical therapy or when LVEF ≤35% and QRS duration ≥130 ms with left bundle branch block (Ezekowitz et al., 2017). However, no recommendation existed for HFrEF patients with QRS <120 ms. In fact, more than 59% of HF patients with reduced EF have a QRS duration of less than 120 ms (Brignole et al., 2013). Clinical efforts are being made to find ways to treat such patients.

In recent years, studies have indicated that renal denervation (RDN) might be a potential therapeutic option for the treatment of patients with severe HF (Davies et al., 2013; Gao et al., 2017). It has been reported that RDN is feasible even for cardiac unstable patients with ventricular tachyarrhythmia and electrical storms (Ukena et al., 2012). RDN can decrease sympathetic activity and result in the restoration of balance between sympathetic and parasympathetic nervous systems (Das, 2013) and reduce ventricular tachyarrhythmia (Ukena et al., 2012).

Our group began to perform RDN surgery on patients with HF in the year 2013. According to the international standard operation methods, the procedure was performed through the femoral artery, under the guidance of a 7F peripheral vascular guide catheter, RDN ablation was performed using a 5F saline perfusion ablation catheter (hda5c090tc, Shanghai Wisegain Medical Devices Co., Ltd.). This kind of 5F saline perfusion ablation catheter is specially designed with different special bend types that can nestle the renal artery steadily according to different vascular shapes. On the one hand, this ablation method can reduce and avoid the damage to the renal artery wall; on the other hand, the provided ablation energy is sufficient to achieve the desired ablation efficacy. So far, RDN ablation has been performed in more than 90 patients at our center, and we have not found any serious complications after RDN treatment.

The implantation rate of implantable cardioverter defibrillators (ICDs) for patients with severe HF has been increasing recently (Gronda et al., 2013), thus indicating the superiority of ICDs over antiarrhythmic drugs in the prevention of sudden cardiac death (SCD) in HF patients (Cappato and Ali, 2017).

In this randomized controlled investigation, we tested the hypothesis that RDN on top of ICD might achieve better clinical results focusing on cardiac function and malignant arrhythmia than ICD alone in HFrEF patients with narrow QRS complexes (QRS duration <120 ms).

## Method and materials

### Study population

Adult patients of either sex with HFrEF were recruited in this study during 17 April 2014 to 22 November 2016. The inclusion criteria were as follows: 1) patients with chronic HF and NYHA classification III or IV; 2) patients aged 18 years and above; 3) patients treated with HF medications according to the guidelines (Chinese society of Cardiology and editorial board of Chinese Journal of Cardiology, 2014), including β-blockers, angiotensin-converting enzyme inhibitor (ACEI), or angiotensin receptor blocker (ARB) plus spironolactone for at least 1 month; 4) patients with left ventricular ejection fraction (LVEF) <40%, assessed using echocardiography; 5) patients with QRS duration <120 ms; and 5) patients identified as candidates for ICD implantation in accordance with the current guidelines.

The exclusion criteria were as follows: 1) renal artery stenosis; 2) estimated glomerular filtration rate (GFR) <30 ml/min/1.73 m^2^; 3) type 1 diabetes mellitus; 4) severe valvular heart disease; 5) pregnant or wish to become pregnant during the study period; 6) acute stage of myocardial infarction or cerebrovascular accident; and 7) systolic blood pressure (SBP) <100 mm·Hg.

### Study design

This is a randomized, open-label, controlled, single-center, feasibility study. Eligible patients were randomized to the RDN plus ICD group or the ICD only group (control group) in a 1:1 ratio. A block random allocation sequence generated by an independent statistician was assigned to the site *via* random envelopes. The primary endpoint was LVEF improvement. We anticipated the increase in LVEF by 5% ± 3% in the RDN plus ICD group compared with the ICD only group. It was estimated that the sample size of each group was 9 (when *α* = 0.05 and power = 90%), so the sample size of each group of was determined as 10.

Blood biochemistry, B-type natriuretic peptide (BNP), echocardiographic indexes, 6-minute walk distance (6MWD), NYHA classification, Holter and 24-h ambulatory blood pressure [SBP and diastolic blood pressure (DBP)], and ICD discharge events were assessed before ICD and 1 year later and up to 3 years post ICD implantation for the ICD only group. For the ICD plus RDN group, the above assessments were performed before the RDN procedure, 1 year later, and up to 3 years after the procedure. All patients were followed up monthly by clinic visit and followed up for up to 3 years.

### Measurement of 24-h ambulatory blood pressure

All patients received 24-h ambulatory blood pressure measurement (instrument model: AND TM2430) before the operation and at 1- and 3-year follow-up after the operation. Blood pressure was measured at 30-min interval during 6:00 a.m. to 10:00 p.m. and 60-min interval during 10:00 p.m. to 6:00 a.m., and variability was analyzed thereafter.

### Echocardiographic assessment

All patients received an exam of echocardiography (instrument model: SONOS7500, IE33, IS80A, IPEQ7c) before the operation and at 1- and 3-year follow-up after the operation. We measured the left atrial diameter, left ventricular end-diastolic diameter, left ventricular end-systolic inner diameter, and septal thickness using a two-dimensional method and measured LVEF using the Simpson method.

### Implantable cardioverter defibrillator implantation

All patients received a single-chamber ICD therapy (Medtronic MAXIMOIIVRD284VRC, St. Jude Current VR 1107-36, St. JudeEpic + VRV-196, Biotronik Lumax340VR, Biotronik Lumax 300VR-T, or Biotronik Lumax 540VR-T DX) according to the current guidelines (Brignole et al., 2013).

### Renal denervation procedure

Patients in the ICD plus RDN group underwent RDN treatment within 1 week after ICD implantation. Patients were treated with an enteric-coated aspirin tablet (300 mg) and clopidogrel (300 mg) before the RDN operation. Heparin (6,000–8,000 U, i.v.) was administered during the operation. We used the standardized protocols of the renal denervation procedure and added more ablation points for each side renal artery to enhance the effectiveness of RDN (Davies et al., 2013). The procedure was performed through the femoral artery. A 7F peripheral guiding catheter was advanced to the renal artery through the femoral artery, and renal arteriography was performed. A 5F saline-perfused radiofrequency ablation catheter (HDA5C090TC, Shanghai Wisegain Medical Devices Co., Ltd.) was then maneuvered within the renal artery. Spiral ablation was conducted with that radiofrequency energy applied to 8–12 W, and temperature was set to 43°C. Each side had 6–10 ablation points with a 0.5 cm of interval in two adjacent points based on the length of the renal artery, and each ablation point lasted for 60 s. We consider that at least 10% of decrease in impedance in one ablation point is successful. Renal arteriography was performed after the RDN procedure.

### Implantable cardioverter defibrillator discharge

ICDs are designed to prevent SCD by converting ventricular arrhythmias into a normal rhythm by the use of antitachycardia pacing (ATP) when ventricular tachycardia attacks or shock therapy when ventricular fibrillation occurs (Kraaier et al., 2013). We defined malignant arrhythmia as a specified diagnosis that needs ATP or shock therapy using an ICD in the two groups. Electrical storm was defined as recurrent ventricular tachycardia or fibrillation occurring three or more times in a 24-h period and requiring electrical cardioversion or defibrillation (shock) therapy (Credner, 1998; Muratore et al., 2008). Patients in both groups were monitored for ICD events up to 3 years after ICD implantation. An ICD event was defined as ICD ATP therapy used when ventricular tachycardia occurred or ICD *in vivo* defibrillation therapy used when ventricular fibrillation occurred. One ICD discharge means an ICD event.

### Statistical analysis

This feasibility study was designed to compare the difference in average percent LVEF using the Simpson method as a change from baseline to 3 years between the ICD plus RDN group and the ICD only group. Ten patients were expected to be enrolled in each group. Continuous data are expressed as mean ± standard deviation. Categorical or dichotomous variables are expressed as percentages. Normality of distribution of all continuous variables was explored by examining skewness, kurtosis, and Q–Q plots. Unpaired Student’s *t*-test or the Mann–Whitney test was used to compare differences in means or mean ranks of variables between the two groups. Paired Student’s *t*-test or the Wilcoxon signed-rank test was used to compare the means or mean ranks of the two related samples (baseline vs. follow-up) as indicated. Fisher’s exact test (rate comparison) was performed to compare proportions. The outcome endpoints were analyzed further utilizing mixed model repeated measures (MMRM) modeling. The MMRM analysis contained terms for treatment group, visit, baseline measurement, and visit by treatment group interaction. SAS v.9.2 software was used for the statistical analyses. A two-sided *p* value of less than 0.05 was considered to indicate statistical significance.

## Results

### Clinical characteristics and biochemical and echocardiographic measurements

Before the operation, the mean [±standard deviation (SD)] age of patients in the ICD plus RDN group and the ICD only group was 67.50 (±13.43) and 64.30 (±11.11) years, respectively, *p* = 0.5686 (Table 1). LVEF was similar between the two groups [31.20% (±3.71%) vs. 31.10% (±3.38%), *p* = 0.9504]. Other baseline features including heart rate, blood pressure (SBP and DBP), 6MWD, NYHA function class, BNP, and other echocardiography indexes were also similar between the two groups (Table 1).

**TABLE 1:** Comparison between the ICD plus RDN group and the ICD only group in clinical characteristics, biochemical, and echocardiography.

	ICD plus RDN group mean (±SD)			ICD only group mean (±SD)			ICD plus RDN group vs. ICD only group *p* value^a^		
	Before operation (*N* = 10)	1 year after operation (*N* = 10)	3 years after operation (*N* = 10)	Before operation (*N* = 10)	1 year after operation (*N* = 10)	3 years after operation (*N* = 10)	Before operation	1 year after operation	3 years after operation
Age/years	67.50 (13.43)			64.30 (11.11)			0.5686	NC	NC
Heart Rate/beats per min	73.30 (15.11)	68.90 (9.29)	68.25 (13.15)	70.80 (13.27)	71.30 (13.72)		0.6989	0.6524	NC
Systolic blood pressure/mmHg	129.60 (10.78)	122.20 (13.28)	116.00 (9.63)	122.60 (10.43)	108.80 (14.29)		0.1573	0.0435	NC
Diastolic blood pressure/mmHg	74.60 (6.75)	70.00 (8.65)	68.50 (10.72)	69.90 (4.77)	65.30 (7.57)		0.0890	0.2125	NC
6MWD/m	126.80 (20.70)	450.50 (25.57)	498.60 (11.26)	126.10 (17.32)	120.60 (13.24)	338.75 (32.76)	0.9355	<0.0001	<0.0001
NYHA cardiac function classification/Grade	3.50 (0.53)	1.50 (0.53)	2.00 (0.00)	3.60 (0.52)	3.20 (0.79)	3.67 (0.58)	0.6733	<0.0001	0.0377
BNP/pg.L^−1^	1,127.99 (822.47)	167.36 (128.49)	795.91 (789.23)	749.22 (411.61)	2,232.29 (2,451.64)	940.64 (819.45)	0.2092	0.0259	0.7833
Left atrial diameter/mm	45.40 (7.38)	43.60 (6.43)	47.00 (6.38)	41.10 (7.61)	44.20 (7.87)	46.00 (10.89)	0.2158	0.8540	0.8443
Left ventricular end-diastolic diameter/mm	65.60 (5.89)	61.90 (9.29)	64.71 (8.67)	67.50 (5.87)	68.60 (5.64)	65.80 (6.38)	0.4794	0.0670	0.8178
Left ventricular end-systolic inner diameter/mm	53.10 (7.53)	46.90 (11.06)	51.29 (10.86)	55.00 (7.29)	56.10 (6.33)	54.60 (9.66)	0.5736	0.0348	0.5980
Left ventricular ejection fraction/%	31.20 (3.71)	39.50 (9.63)	41.57 (9.62)	31.10 (3.38)	31.20 (4.52)	31.40 (8.14)	0.9504	0.0285	0.0842
Septal thickness/mm	9.30 (2.11)	8.60 (2.95)	9.43 (1.90)	8.30 (1.64)	8.70 (1.70)	8.00 (2.45)	0.2518	0.9271	0.2804

a *t*-test.

NC, not calculated; N, number of population analysis set; n, number of patients; SD, standard deviation; ICD, implantable cardioverter defibrillator; RDN, renal denervation; 6MWD, 6-min walk distance; NYHA, New York Heart Association; BNP, B-type natriuretic peptide.

**FIGURE 1:**
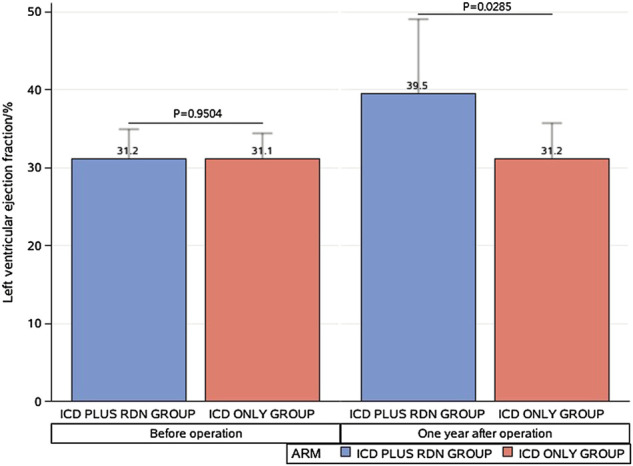
Comparison between the implantable cardioverter defibrillator (ICD) plus renal denervation (RDN) group and the ICD only group in left ventricular ejection.

One year after the operation, the mean LVEF (%) was significantly higher in the ICD plus RDN group than in the ICD alone group (39.50% ± 9.63% vs. 31.20% ± 4.52%, *p* = 0.0285) and tended to be higher at 3 years after the operation (41.57% ± 9.62% vs. 31.40% ± 8.14%, *p* = 0.0842) (Table 1; Figure 1). The ICD plus RDN group also shows more improvement than the ICD alone group in other echocardiography indicators, including left atrial diameter, left ventricular end-diastolic diameter, left ventricular end-systolic inner diameter, and septal thickness (Tables 2, 3).

**TABLE 2:** Comparison among visits of the ICD plus RDN group in clinical characteristics, biochemical, and echocardiography.

	ICD plus RDN group mean (±SD)			*p* value^a^		
	Before operation (*N* = 10)	1 year after operation (*N* = 10)	3 years after operation (*N* = 10)	Before operation vs. 1 year after operation	Before operation vs. 3 years after operation	1 year after operation vs. 3 years after operation
Heart Rate/beats per min	73.30 (15.11)	68.90 (9.29)	68.25 (13.15)	0.4431	0.5709	0.9175
Systolic blood pressure/mmHg	129.60 (10.78)	122.20 (13.28)	116.00 (9.63)	0.1882	0.0492	0.4171
Diastolic blood pressure/mmHg	74.60 (6.75)	70.00 (8.65)	68.50 (10.72)	0.2017	0.2182	0.7879
6MWD/m	126.80 (20.70)	450.50 (25.57)	498.60 (11.26)	<0.0001	<0.0001	0.0016
NYHA classification/grade	3.50 (0.53)	1.50 (0.53)	2.00 (0.00)	<0.0001	<0.0001	0.0150
BNP/pg.L^−1^	1,127.99 (822.47)	167.36 (128.49)	795.91 (789.23)	0.0049	0.4688	0.1497
Left atrial diameter/mm	45.40 (7.38)	43.60 (6.43)	47.00 (6.38)	0.5682	0.6493	0.2988
Left ventricular end-diastolic diameter/mm	65.60 (5.89)	61.90 (9.29)	64.71 (8.67)	0.3016	0.8046	0.5375
Left ventricular end-systolic inner diameter/mm	53.10 (7.53)	46.90 (11.06)	51.29 (10.86)	0.1602	0.6887	0.4303
Left ventricular ejection fraction/%	31.20 (3.71)	39.50 (9.63)	41.57 (9.62)	0.0263	0.0290	0.6686
Septal thickness/mm	9.30 (2.11)	8.60 (2.95)	9.43 (1.90)	0.5495	0.8994	0.5250

a *t*-test.

NC, not calculated; N, number of population analysis set; n, number of patients; SD, standard deviation; ICD, implantable cardioverter defibrillator; RDN, renal denervation; 6MWD, 6-min walk distance; NYHA, New York Heart Association; BNP, B-type natriuretic peptide.

**TABLE 3:** Comparison among visits of the ICD only group in clinical characteristics, biochemical, and echocardiography.

	ICD only group mean (±SD)			*p* value^a^		
	Before operation (*N* = 10)	1 year after operation (*N* = 10)	3 years after operation (*N* = 10)	Before operation vs. 1 year after operation	Before operation vs. 3 years after operation	1 year after operation vs. 3 years after operation
Heart Rate/beats per min	70.80 (13.27)	71.30 (13.72)		0.9349	NC	NC
Systolic blood pressure/mmHg	122.60 (10.43)	108.80 (14.29)		0.0239	NC	NC
Diastolic blood pressure/mmHg	69.90 (4.77)	65.30 (7.57)		0.1215	NC	NC
6MWD/m	126.10 (17.32)	120.60 (13.24)	338.75 (32.76)	0.4355	<0.0001	0.0005
NYHA cardiac function classification/Grade	3.60 (0.52)	3.20 (0.79)	3.67 (0.58)	0.1964	0.8514	0.3678
BNP/pg.L^−1^	749.22 (411.61)	2,232.29 (2,451.64)	940.64 (819.45)	0.0901	0.5498	0.1576
Left atrial diameter/mm	41.10 (7.61)	44.20 (7.87)	46.00 (10.89)	0.3823	0.3251	0.7181
Left ventricular end-diastolic diameter/mm	67.50 (5.87)	68.60 (5.64)	65.80 (6.38)	0.6743	0.6156	0.4003
Left ventricular end-systolic inner diameter/mm	55.00 (7.29)	56.10 (6.33)	54.60 (9.66)	0.7228	0.9295	0.7214
Left ventricular ejection fraction/%	31.10 (3.38)	31.20 (4.52)	31.40 (8.14)	0.9559	0.9402	0.9514
Septal thickness/mm	8.30 (1.64)	8.70 (1.70)	8.00 (2.45)	0.5988	0.7803	0.5264

a *t*-test.

NC, not calculated; N, number of population analysis set; n, number of patients; ICD, implantable cardioverter defibrillator; RDN, renal denervation; 6MWD, 6-min walk distance; NYHA, New York Heart Association; BNP, B-type natriuretic peptide.

With consideration for potential interaction of treatment group and time visit, we performed MMRM analysis to evaluate whether there is an overall significant difference between the groups. An overall MMRM *p* value of 0.0306 was found on LVEF, showing a superior efficacy for the patients treated with ICD plus RDN in terms of LVEF (Table 4).

**TABLE 4:** Overall test between the ICD plus RDN group and the ICD only group in clinical characteristics, biochemical, and echocardiography over all visits.

	ICD plus RDN group mean (±SD)			ICD only group mean (±SD)			Overall *p* value^a^ ICD plus RDN group vs. ICD only group
	Before operation (*N* = 10)	1 year after operation (*N* = 10)	3 years after operation (*N* = 10)	Before operation (*N* = 10)	1 year after operation (*N* = 10)	3 years after operation (*N* = 10)	
Heart Rate/beats per min	73.30 (15.11)	68.90 (9.29)	68.25 (13.15)	70.80 (13.27)	71.30 (13.72)		0.9903
Systolic blood pressure/mmHg	129.60 (10.78)	122.20 (13.28)	116.00 (9.63)	122.60 (10.43)	108.80 (14.29)		0.0356
Diastolic blood pressure/mmHg	74.60 (6.75)	70.00 (8.65)	68.50 (10.72)	69.90 (4.77)	65.30 (7.57)		0.0616
6MWD/m	126.80 (20.70)	450.50 (25.57)	498.60 (11.26)	126.10 (17.32)	120.60 (13.24)	338.75 (32.76)	<0.0001
NYHA cardiac function classification/grade	3.50 (0.53)	1.50 (0.53)	2.00 (0.00)	3.60 (0.52)	3.20 (0.79)	3.67 (0.58)	<0.0001
BNP/pg.L^−1^	1,127.99 (822.47)	167.36 (128.49)	795.91 (789.23)	749.22 (411.61)	2,232.29 (2,451.64)	940.64 (819.45)	0.0940
Left atrial diameter/mm	45.40 (7.38)	43.60 (6.43)	47.00 (6.38)	41.10 (7.61)	44.20 (7.87)	46.00 (10.89)	0.7209
Left ventricular end-diastolic diameter/mm	65.60 (5.89)	61.90 (9.29)	64.71 (8.67)	67.50 (5.87)	68.60 (5.64)	65.80 (6.38)	0.3108
Left ventricular end-systolic inner diameter/mm	53.10 (7.53)	46.90 (11.06)	51.29 (10.86)	55.00 (7.29)	56.10 (6.33)	54.60 (9.66)	0.2174
Left ventricular ejection fraction/%	31.20 (3.71)	39.50 (9.63)	41.57 (9.62)	31.10 (3.38)	31.20 (4.52)	31.40 (8.14)	0.0306
Septal thickness/mm	9.30 (2.11)	8.60 (2.95)	9.43 (1.90)	8.30 (1.64)	8.70 (1.70)	8.00 (2.45)	0.3761

a Mixed model repeated measures (MMRM) analysis was performed with consideration in interaction of treatment group and time visit point.

Note 2: NC, not calculated; N, number of population analysis set; n, number of patients; SD, standard deviation; ICD, implantable cardioverter defibrillator; RDN, renal denervation; 6MWD, 6-min walk distance; NYHA, New York Heart Association; BNP, B-type natriuretic peptide.

As shown in Table 4, the superior efficacy of combined treatment of ICD plus RDN was indicated by the higher SBP (MMRM *p* = 0.0356), longer 6MWD (*p* < 0.0001), and lower NYHA classification (*p* < 0.0001) during follow-up.

### Implantable cardioverter defibrillator discharge

In the ICD plus RDN group, three patients experienced 31 times ICD discharge after the ICD operation (27 times in one patient and two times each in the other two patients) before the RDN operation. In the ICD only group, one patient experienced 43 times ICD discharge after the ICD operation. In the ICD plus RDN group, two patients reported one ICD discharge each at 1 year after the operation, one patient reported one ICD discharge at 3 years after the operation. In the ICD only group, five patients reported 40 ICD discharge events at 1 year after the ICD operation. Among them, three patients experienced frequent electric storms (5, 9, and 24 times). Another two ICD discharge events were reported in one patient at 3 years after the operation (Table 5).

**TABLE 5:** ICD discharge and etiology in the ICD plus RDN group and ICD only group.

Group	Number	Etiology	Before RDN operation	1 year after RDN/ICD operation	3 years after RDN/ICD operation
ICD plus RDN group	01	DCM	0	0	—
	02	HTN	0	0	0
	03	OMI	0	0	0
	04	OMI	0	0	0
	05	ICM	27 (24ATP, 3SHOCK)	0	0
	06	OMI	2 (2ATP)	0	—
	07	HTN	2 (2SHOCK)	1 (1ATP)	—
	08	DCM	0	0	0
	09	OMI	0	1 (1ATP)	0
	10	OMI	0	0	1 (1SHOCK)
	Subtotal		31	2	1
ICD only group	11	ICM	0	1 (1SHOCK)	—
	12	DCM	0	0	—
	13	HTN	0	5 (5ATP)	—
	14	OMI	0	0	0
	15	HTN	0	0	—
	16	OMI	0	0	—
	17	OMI	0	0	0
	18	OMI	0	1 (1ATP)	0
	19	DCM	0	9 (6ATP, 3SHOCK)	2 (1ATP, 1SHOCK)
	20	DCM	43 (2ATP,41SHOCK)	24 (1ATP, 23SHOCK)	0
	Subtotal		43	40	2

DCM, dilated cardiomyopathy; HTN, hypertension; OMI, old myocardial infarction; ICM, ischemic cardiomyopathy; ICD, implantable cardioverter defibrillator; RDN, renal denervation.

### Preoperative and postoperative medications between the two groups

Before the operation, all patients in both groups were treated with a combination therapy of spironolactone, ACEI/ARB, β-blocker, and loop diuretics. One year after the RDN operation, the use of loop diuretics was significantly reduced in the ICD plus RDN group at 1 year (20% vs. 80%, *p* = 0.0230) and at 3 years post operation (28.57% vs. 60%, *p* = 0.5581) (Table 6).

**TABLE 6:** Medications used at preoperative and postoperative in the ICD plus RDN group and ICD only group.

Group	Patient number	Loop diuretic	Before operation		β-blocker	Loop diuretic	1 year after operation		β-blocker	Loop diuretic	3 years after operation		β-blocker
			Spironolactone ACEI/ARB				Spironolactone ACEI/ARB				Spironolactone ACEI/ARB		
ICD plus RDN group	1	Y	Y	Y	Y	N	N	N	Y	-	-	-	-
	2	Y	Y	Y	Y	N	N	Y	Y	N	N	Y	Y
	3	Y	Y	Y	Y	Y	Y	Y	Y	Y	Y	Y	Y
	4	Y	Y	Y	Y	Y	Y	Y	Y	Y	Y	Y	Y
	5	Y	Y	Y	Y	N	N	Y	Y	N	N	Y	Y
	6	Y	Y	Y	Y	N	N	Y	Y	-	-	-	-
	7	Y	Y	Y	Y	N	Y	Y	Y	-	-	-	-
	8	Y	Y	Y	Y	N	Y	N	Y	N	Y	Y	Y
	9	Y	Y	Y	Y	N	N	Y	Y	N	N	Y	Y
	10	Y	Y	Y	Y	N	Y	N	Y	N	N	Y	Y
	Subtotal n (%)	10 (100%)	10 (100%)	10 (100%)	10 (100%)	2 (20%)	5 (50%)	7 (70%)	10 (100%)	2 (28.57%)	3 (42.86%)	7 (100%)	7 (100%)
ICD only group	1	Y	Y	Y	Y	Y	Y	Y	Y	-	-	-	-
	2	Y	Y	Y	Y	Y	Y	Y	Y	-	-	-	-
	3	Y	Y	Y	Y	Y	Y	Y	Y	-	-	-	-
	4	Y	Y	Y	Y	Y	Y	Y	Y	Y	Y	Y	Y
	5	Y	Y	Y	Y	Y	Y	Y	Y	-	-	-	-
	6	Y	Y	Y	Y	Y	Y	N	Y	-	-	-	-
	7	Y	Y	Y	Y	N	N	N	Y	N	N	N	Y
	8	Y	Y	Y	Y	N	Y	Y	Y	N	Y	Y	Y
	9	Y	Y	Y	Y	Y	Y	Y	Y	Y	Y	Y	Y
	10	Y	Y	Y	Y	Y	Y	Y	Y	Y	Y	Y	Y
	Subtotal n (%)	10 (100%)	10 (100%)	10 (100%)	10 (100%)	8 (80%)	9 (90%)	8 (80%)	10 (100%)	3 (60%)	4 (80%)	4 (80%)	5 (100%)
	*p* Value					0.0230				0.5581			

Note: ICD, implantable cardioverter defibrillator; RDN, renal denervation; ACEI, angiotensin-converting enzyme inhibitor; ARB, angiotensin receptor blocker.

### Rehospitalization and cardiogenic mortality

All patients were alive at the 1 year follow-up. Three years after the operation, seven patients in the ICD plus RDN group and five patients in the ICD only group were alive. The rehospitalization rate was lower in the ICD plus RDN group than in the ICD only group during the study period (30% vs. 100%, respectively). The cardiogenic mortality rate was 0% in the ICD plus RDN group and 50% in the ICD only group (Table 7).

**TABLE 7:** Rehospitalization after operation and cardiogenic mortality in the ICD plus RDN group and ICD only group.

Group	Patients number	Times of rehospitalization	Rehospitalization	Status of 3 years after operation	Death reason	Cardiogenic mortality
ICD plus RDN group	01	0	N	Decease	Hepatic metastasis of gastric cancer	N
	02	0	N	Alive	—	—
	03	3	Y	Alive	—	—
	04	0	N	Alive	—	—
	05	0	N	Alive	—	—
	06	0	N	Decease	Advanced lung cancer	N
	07	14	Y	Decease	Acute exacerbation of chronic renal failure	N
	08	3	Y	Alive	—	—
	09	0	N	Alive	—	—
	10	0	N	Alive	—	—
	Subtotal	20	30%	—	—	0
ICD only group	11	8	Y	Decease	Acute onset of chronic heart failure	Y
	12	7	Y	Decease	Cardiogenic shock	Y
	13	1 (be hospitalized in another hospital)	Y	Decease	Acute onset of chronic heart failure	Y
	14	2	Y	Alive	—	—
	15	2	Y	Decease	Acute onset of chronic heart failure	Y
	16	7	Y	Decease	Acute onset of chronic heart failure	Y
	17	1 (be hospitalized in another hospital)	Y	Alive	—	—
	18	4	Y	Alive	—	—
	19	10	Y	Alive	—	—
	20	3	Y	Alive	—	—
	Subtotal	45	100%	—	—	50%
	*p* value		0.0031			0.0325

Note: ICD, implantable cardioverter defibrillator; RDN, renal denervation.

## Discussion

This randomized controlled pilot investigation provided evidence that additional RDN therapy on top of ICD is superior to ICD only for severe chronic HF patients with narrow QRS complexes (QRS duration <120 ms). RDN therapy resulted in more significant symptomatic alleviation and further improved outcome, in that RDN therapy after ICD implantation resulted in significant improvement in SBP, 6MWD, NYHA classification, and LVEF, as compared to ICD only therapy (Table 4). Furthermore, there was marginal decrease in ICD discharge at 1 year after the operation, and this trend is stable in the following 3 years (Table 5). In terms of medication therapy, obvious decreased use of loop diuretics was noted. In addition, the combined treatment is also associated with lower rehospitalization and cardiogenic death rates (Tables 6 and 7).

It was known that the main pathogenesis of chronic HF is the overactivation of the sympathetic nervous and renin–angiotensin systems. The sympathetic nervous system is markedly activated in patients with chronic heart disease (Rubinsztajn, 2001). A recent study showed that RDN can improve heart function by inhibiting the renin–angiotensin system and resisting left ventricular remodeling in a rapid pacing pig HF model (Xie et al., 2014). In two studies performed on norepinephrine-induced cardiomyopathy and HF models in rats, it was found that RDN can inhibit transforming growth factor-β1, MMP2, collagen I, and inflammatory cytokines CRP and TNF-α. Compared with the sham operation group, RDN can inhibit sympathetic hyperactivity and rebalance the renin–angiotensin–aldosterone system (RAAS) axis, thus inhibiting cardiorenal fibrosis, through multiple ways including downregulation of aldosterone and ACE II 1 receptor and upregulation of ACE II 1-7/MAS receptor (Liu et al., 2015a; Liu et al., 2015b). In another study, RDN was found to improve the cardiac function of rats with HF by modulating the expression of β-adrenergic receptor (Zheng et al., 2016).

Results from the REACH-Pilot study (Davies et al., 2013) showed that RDN was effective and safe for HF patients. The results suggested improvements in both symptoms and exercise performance post RDN. Another randomized controlled pilot study (Chen et al., 2017) demonstrated that catheter-based renal denervation using a saline-irrigated catheter could be safely applied to the treatment of HF and can improve the cardiac systolic function and patients’ quality of life. The LVEF, 6MWD, and NYHA class in the RDN group were significantly improved compared with those in the control group over 6 months follow-up.

In our study, ICD was implanted in all severe HF patients with narrow QRS wave (<120 ms) and LVEF <40% according to the guidelines (Brignole et al., 2013). In the ICD plus RDN group, 6MWD increased by 255.3%, NYHA cardiac function class decreased by 57.1%; BNP decreased by 85.2%, and LVEF at echocardiography increased by 26.6% at 1 year after the operation.

When patients were followed up, 6MWD increased by more than 292.2%, NYHA classification decreased by 42.9%, and LVEF derived from echocardiography increased by 33.2% at 3 years after the operation (Table 2). In contrast, in the ICD only group, there were no significant statistical differences observed in 6MWD, NYHA function class, BNP, and LVEF at 1 and 3 years after the ICD operation except increased 6MWD at 3 years after the ICD operation (Table 3). Comparison between the two treatment groups 1 and 3 years after the operation showed that the ICD plus RDN group had significant improvement than the ICD only group in terms of 6MWD and NYHA cardiac function class (Table 4). The results suggested that the cardiac function and exercise capacity in patients of severe chronic HF with narrow QRS wave were improved after RDN treatment on top of ICD. Results from medication analysis indicated that combined RDN and ICD treatment decreased the need for diuretic medication (Table 6). No hypotension and deteriorated renal function were observed before and 1 and 3 years after RDN in the ICD plus RDN group, indicating the safety of RDN. Overall, our study results are in line with above-mentioned experimental and clinical studies, in that RDN is beneficial in the setting of HF, possibly *via* decreasing the sympathetic activity.

Results from primary prevention of SCD studies [MADITI, MADIT II, and MUSTT (Prystowsky and Nisam, 2000; Moss et al., 1996; Moss et al., 2006)] and secondary prevention of SCD studies [AVID, CIDS, and CASH (None, 1997; Bella, 2000; Kuck et al., 2000)] had demonstrated that the survival benefit of an ICD in patients with life-threatening ventricular arrhythmias, made progress in the prevention of SCD. With the exception of the ICD, there are few effective strategies for the prevention and treatment of SCD (Darbar, 2007). However, despite the unequivocal clinical benefit, ICDs are known to have a complex psychosocial impact on the patients (Jacob et al., 2012). ATP and shock therapies (both appropriate and inappropriate), especially the electrical storms, were related to psychological trauma and deterioration of ventricular function (Chinese society of Cardiology and editorial board of Chinese Journal of Cardiology, 2014; Poole et al., 2016; Ukena et al., 2016).

Moreover, the sympathetic nervous system was viewed as a potent stimulus for ventricular tachyarrhythmia and SCD (Podrid et al., 1990). It is believed that the effectiveness of RDN is because of decreased sympathetic activity and because of the restoration of balance between the sympathetic and parasympathetic nervous systems (Das, 2013). Following RDN, it was found that RDN is feasible even in cardiac unstable patients, abd ventricular tachyarrhythmia was significantly reduced in these patients (Ukena et al., 2012).

Further study proved that RDN could effectively reduce the arrhythmic burden for recurrent ventricular arrhythmias (Ukena et al., 2016). In our study, ICD discharge was a marginal decrease in the ICD plus RDN group as compared to the ICD only group (Table 5). Thus, our study presented more favorable clinical results of ICD plus RDN strategies as compared to ICD only in HF patients with reduced LVEF and narrow QRS complexes.

## Conclusion

Results from this feasibility study indicate that RDN on top of ICD implantation is superior to ICD alone in improving cardiac function and clinical outcome in HF patients with reduced LVEF and narrow QRS complexes (QRS duration <120 ms). Large-sized randomized controlled trials are warranted in the future to validate the results of this pilot feasibility investigation.

## Limitations

The small number of patients and single-center study design were the limitations of this RCT study. Future studies with more patients from multiple centers are warranted to validate the effect and safety of RDN in the treatment of HFrEF patients.

## Data Availability

The raw data supporting the conclusion of this article will be made available by the authors, without undue reservation.
